# Symmetrical choices and biased confidence during uncertain personality trait judgments

**DOI:** 10.1371/journal.pone.0312858

**Published:** 2024-10-31

**Authors:** Lorenz Weise, Barbara Drüke, Siegfried Gauggel, Verena Mainz

**Affiliations:** Institut für Medizinische Psychologie und Medizinische Soziologie, Uniklinik der RWTH Aachen, Aachen, Germany; Julius-Maximilians-Universität Würzburg: Julius-Maximilians-Universitat Wurzburg, GERMANY

## Abstract

While great methodological strides have been made in the area of decision making research, decisions that rely on subjective stimuli, such as personality traits, still pose a challenge for researchers, partly because it is difficult to define a standard of accuracy for such choices–they lack a “ground truth”. In studies on value-based decisions, this same problem has been circumvented by comparing uncertain subjective decisions against a separately assessed judgment of value–a “standard”. Here we apply this method in a task of verbal personality trait judgment, and show how a separately assessed standard judgment can be used to precisely control stimulus presentation and analyze subjective personality choices via the method of reverse correlation. Per trial, a series of quasi-randomly sampled adjectives was shown, which participants categorized as more descriptive of either themselves of another person well known to them. Participants also indicated their confidence in the response. Each trial’s difficulty was controlled based on the previously assessed standard judgment. Analysis of the behavioral data shows several decision-general properties during these personality judgments, such as symmetrical choices, steeper choice functions for confident trials, and a positive evidence bias during confidence judgment. We discuss how these findings can shed light on the cognitive processes involved in personality perception. The task and results together may help bridge the gap between research on complex, social forms of judgment and findings on more basic decision processes.

## Introduction

Psychological researchers have been interested in the processes involved in choices and decision making for a long time. Some of the earliest psychological experiments involved choice tasks [1], such as deciding which of two weights is heavier or which of two light patches is brighter. Metacognitive evaluations of choices, such as the subjective confidence about a judgment, have a similarly long history [2]. It is understandable that choices have attracted such an interest in research on human cognition, as it was recognized from the earliest days of psychological science that they are the primary evidence for human volition [3].

It is safe to say that experimental psychology has since then made great progress in uncovering the cognitive basis of choices and decisions. On the methodological side, researchers have developed sophisticated methods of tightly controlling and dynamically adjusting decision stimuli and their difficulty [e.g. 4–6], creating uncertain judgment situations in which participants have to try their best to respond accurately. This has allowed precise laboratory investigations into perceptual decision making and metacognition. In the area of data analysis, methods such as reverse correlation [7], whereby participants’ responses are related back to fluctuations in stimulus properties, have uncovered which aspects of decision stimuli influence choices and confidence [e.g. 8–10].

Formal analysis and quantitative modelling of choice behavior have shed light on the kind of underlying computational processes that might be at play during categorization of motion direction [11], stimulus color [12], relative brightness [10] and many more. Furthermore, precise experimental control and sophisticated methods of data analysis have allowed bridging the gap between postulated models of choice and confidence on the one hand, and neurobiological processes on the other [13–15].

It is no coincidence that in a discussion on methodological advances in the study of decision making, studies on perceptual decisions feature heavily. Sensory stimuli, such as moving dots, color patches, auditory signals and other physical quantities can often be controlled with great precision. This in turn makes them ideal signals for quantitative methods such as control and dynamic adjustment of difficulty, reverse correlation of responses to stimuli, or quantitative modelling of behavioral data. One of the central enabling factors of these methods is the fact that for physical stimuli, there is a *ground truth*: wavelength in the case of color judgments; position on the screen in the case of position or motion judgments; luminance in the case of judging light patches, etc. This ground truth allows defining the accuracy of a decision by comparing it to the participant’s response, and it also allows controlling decision difficulty by using the relationship between the physical stimulus properties and the probability of responding accurately in order to adjust the stimulus in the direction of correct or incorrect responding. This kind of ground truth is obvious for many perceptual decisions, but absent for other types of choices, such as affective-, value-based or personality judgments (“subjective” judgments).

At the same time, it is clear that many of our everyday decisions do not directly operate on sensory stimuli and their internal representations. Whether it is deciding to buy a house or judge a person’s mood, choices often depend on a multitude of signals that are not perceptual in nature, and especially in social contexts, these signals are often transferred to us verbally, via text or speech. Thus, while sensory stimuli can be quantified and controlled precisely, verbal stimuli allow the investigation of many more complex types of decisions. One such decision is the judgment of personality traits, which has received considerable attention in the area of personality psychology [16]. In tasks of personality trait judgment, participants are often presented with trait adjectives (e.g. *intelligent*, *mean*, *warm*, …) and have to indicate whether the adjective is descriptive of a target person (e.g. the participant him-/herself, somebody else known to the participant, a famous person, etc.).

The lack of a simple ground truth for such personality judgments, but also value-based and other decisions, is a pervasive methodological problem when investigating these subjective choices [17,18]. Different ways of conceptualizing the accuracy of such decisions, by finding some kind of *standard* to compare responses to, have been put forward: an agreement between the judge and the person being judged, a consensus between several judges, or the ability of the judgment to predict behavior [16]. Another type of standard has been used in studies on value-based decision making, by including within the study and for each participant a separate assessment of value for every stimulus used in the choice task [19,20]. For example, [19] report a value-choice task in which participants had to choose which of two snacks they wanted to consume more. A separate rating how much the participant would pay for each item in isolation served as the standard against which the two-alternative choices could be compared.

This solution of gathering a separate *standard rating* of decision stimuli for each participant is promising because it allows many of the experimental methods discussed earlier in the context of perceptual stimuli: the standard rating can be used to create uncertain decision trials, in which the stimuli presented for choice do not clearly favor one response over the other according to the participant’s standard. The degree of uncertainty in the response, and thus the difficulty of the choice, can be manipulated in a graded manner, similar to titration methods in psychophysical tasks [21]. Furthermore, responses in these uncertain choice trials can be correlated back to fluctuations in the initial standard rating of the participant, similar to reverse correlation methods often used in tasks of perceptual decision making, in order to identify how these stimulus fluctuations are related to choice and confidence [10].

The present work implements this method of individualized standard ratings of choice stimuli in a task of two-alternative personality trait judgment using verbal stimuli. Per trial, a sequence of four personality-describing adjectives is presented visually, which the participant has to categorize as either more self-descriptive or other-descriptive. The “other” is a person chosen by the participant. Furthermore, participants indicate their confidence for each choice. Varying forms of this self-other judgment task have been employed previously [22,23]. Before the start of the choice task, each participant first gives two “definitive” ratings for each adjective, indicating how well the adjective describes the participant him-/herself, and how well it describes the other person. This standard rating is used during the trait judgment task to create uncertain choice trials by showing sequences of words which overall could favor the participant or the other person to different degrees. Using this method, we create choice situations with a difficulty–in terms of choice accuracy–of around 70%. Furthermore, we apply reverse correlation by relating choices and confidence judgments back to the fluctuations of word ratings in the preceding trial.

We evaluate the behavioral data with two goals. First, we check whether our method of using the participant’s standard ratings for experimental stimulus control works, by evaluating whether a difficulty of around 70% was achieved, whether participants indeed felt partly unsure about their trait judgments, and whether the accuracy–as operationalized using the standard word ratings–corresponds to participants’ subjective sense of correctness of their uncertain choices.

Second, we investigate whether typical, domain-general markers of choices and metacognitive judgments often obtained in perceptual decision tasks can be replicated in this personality trait judgment task. We consider three general patterns of behavioral results reported in the literature:

### Steeper choice functions for confident trials

When participants categorize a physical stimulus, the relationship between stimulus strength and choice probability can be expressed as a curve, the psychometric function. It is usually found that in those trials in which the participant is confident about their choice, this function is steeper compared to those trials in which the participant is not confident [24], indicating an increased sensitivity of responses to the decision stimulus.

*Symmetrical choices*: in two-alternative choices about stimuli that contain separable information relating to both alternatives, it has been found that both the information in favor of the chosen alternative and information in favor of the non-chosen alternative are factored into the decision [e.g. 8–10]. For example, when a cloud of moving dots is categorized as moving up or moving down, movement in the chosen direction positively affects choices, while movement in the opposite direction negatively affects choices [10]. This effect has also been referred to as a “balance-of-evidence rule” [25].

### Decision-congruent confidence

While primary choices are affected both by information for and against the chosen option, it has been found that confidence in the previous choice is selectively affected by information in favor of the chosen option. Thus, when we decide the cloud of dots is moving up, our confidence in this choice is affected by the amount of up-ward movement in the stimulus, while the down-ward movement appears to be discounted or ignored [10,14,26].

## Methods

### Participants

Twenty-eight current or former students took part (age: M = 23 years, range = 18–30; 50% female) and were paid 15€ for their participation. All participants gave written informed consent. The study was approved by the local ethics committee of the university hospital of the RWTH Aachen University (EK 288/18). The data of one participant had to be removed because that person performed the task incorrectly and gave the same response on all trials. Participants were recruited between 01-12-2018 and 31-05-2019.

### Procedure

**Fig 1:**
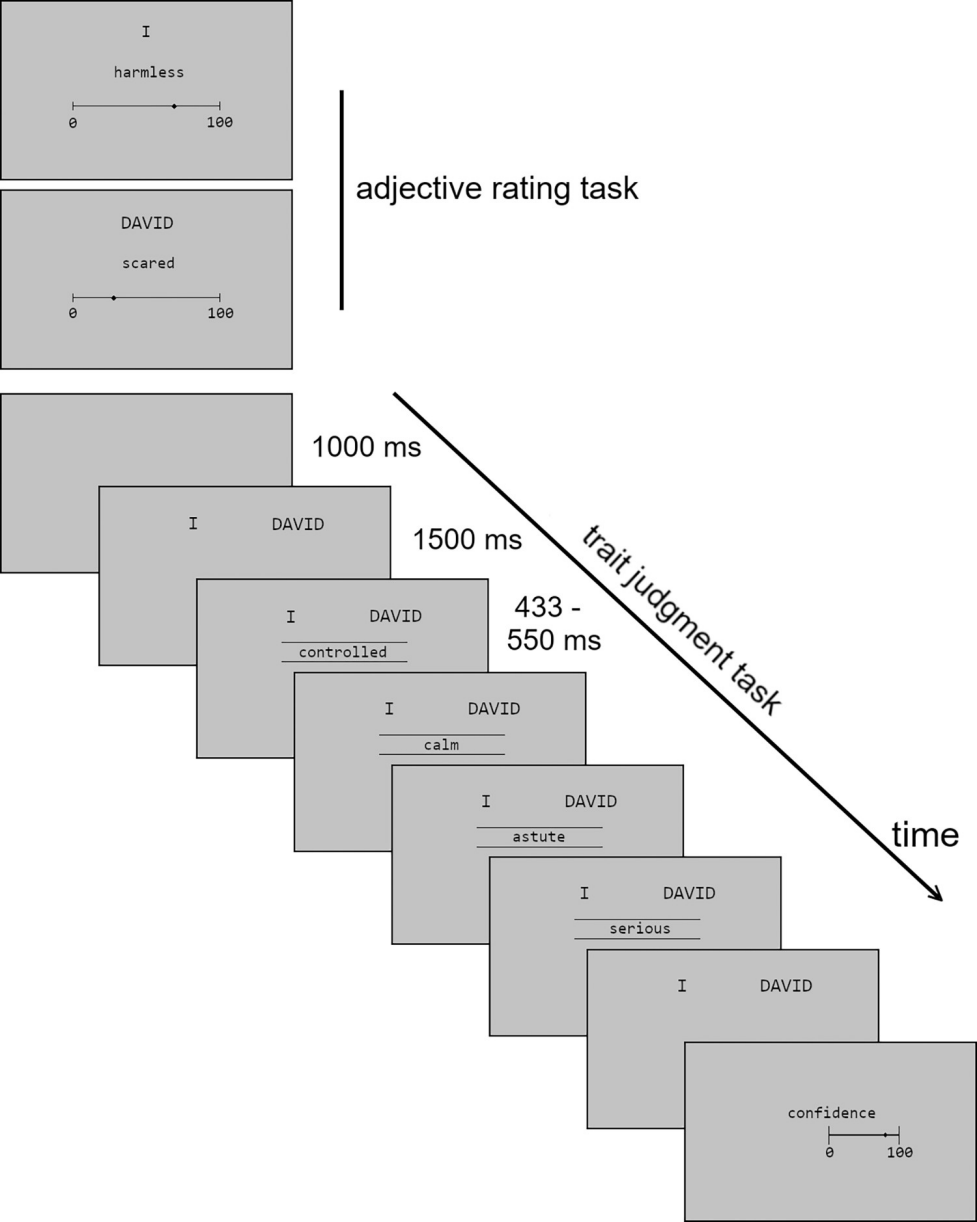
Illustration of the task. The first two images illustrate the initial adjective rating task, once w.r.t. the participant, and once w.r.t. the other. The last sequence of images illustrates one trial of the trait judgment task. First, the two choice alternatives are displayed (“David” is used as an example for an other’s name). Then, a sequence of four words is displayed consecutively, each word displayed between 433 and 550 ms. Next, the choice has to be indicated via mouse button. Lastly, the confidence has to be indicated on a scale of 0 to 100. There was no time limit for indicating choice and confidence. Word stimuli and choice alternative “I” were displayed in German during the actual task.

Participants were seated in front of a laptop (Lenovo Yoga 720-13IKB, 13.3 inch screen diagonal) at about 50 cm distance from the screen. The instructions asked the participant to type in the name of another person well known to them (*the other* from here on). The other should be similar to the participant in some aspects, but different in others. Next came the *adjective rating task*, in which 104 personality-describing adjectives were shown in two randomized blocks: using a visual analog scale (0–100), the participant once rated how well each adjective describes him-/herself (*self rating*), and once how well it describes the other (*other rating*; see Fig 1). These ratings serve as the standard for the *trait judgment task*.

Next, participants filled in a demographic questionnaire as well as the Inclusion of Other in the Self scale [27], measuring how close the other is to the participant. Finally, participants performed the trait judgment task (see the following sections).

### Trait judgment task

In the trait judgment task, participants were presented with a series of four personality-describing adjectives per trial. For each trial’s word sequence, the participant decided whether the group of words better described her-/himself or the other. The response was provided after the last word using the left- and right mouse button. The instructions emphasized that the decision should be based on all four adjectives, and that after their decision, they would be prompted to indicate how confident they were about their previous choice, on a scale from 0 to 100.

The four adjectives per trial were presented centrally on the screen. The word “I” and the other’s name were displayed above the adjectives, offset to the left and right. The side of the two words on the screen was always spatially congruent with the mouse buttons used to give the corresponding response, but which side corresponded to the self-/other response was counterbalanced across participants. Because some words take more time to be recognized than others, the display time of each adjective was set according the predicted recognition time for that word, based on a German corpus of words published along with their lexical decision times [28]. From this corpus, a linear model was derived predicting lexical decision times from the number of letters, syllables and word frequency as well as all interactions. We then used this model to predict lexical decision time for each of our word stimuli, and presented each word for its predicted lexical decision time minus 117 ms, results ranging between 433 and 550 ms. This method resulted in rapid but readable presentation.

After participants had given their response and entered their confidence, a 1000 ms inter-trial interval with a blank screen followed, after which the names were shown for 1500 ms to let the participant prepare for the next decision trial.

Twenty-seven out of the 28 participants completed a total of 306 trials, one participant completed 240 trials. In half of the trials, the adjectives were selected so as to be perceived as more descriptive of the participant according to his/her standard ratings (*self condition*), in the other half they were selected to be perceived as more descriptive of the other person (*other condition;* for details see *per-trial adjective selection*). The order of conditions was randomized. Every 50 trials there was a self-terminated break. The initial rating task took about 5–10 minutes, the main trait judgment task took around 40 minutes.

### Stimulus material

The adjectives were selected from the Aachen List of Trait Words, a pool of German adjectives along with various word properties recently compiled to describe personalities [29]. We aimed to exclude the most strongly valenced adjectives (*aggressive*, *brutal*, *happy*, *honest*) because these often elicit stereotyped personality judgments. To this end, only adjectives with an absolute valence rating of 1.6 or less in the validation study were included. Examples of the most strongly valenced items that were retained are *fearful*, *mocking*, *orderly* and *spontaneous*. Furthermore, rarely used adjectives were excluded (log10 word frequency above 0.5). Examples of the least common items retained are *grimmig* (*grim*) and *nachlässig* (*negligent*). We only included adjectives that were less than 14 letters in length. Finally, eight further adjectives were excluded because they were found to be difficult to rate during piloting.

### Per-trial adjective selection

**Fig 2:**
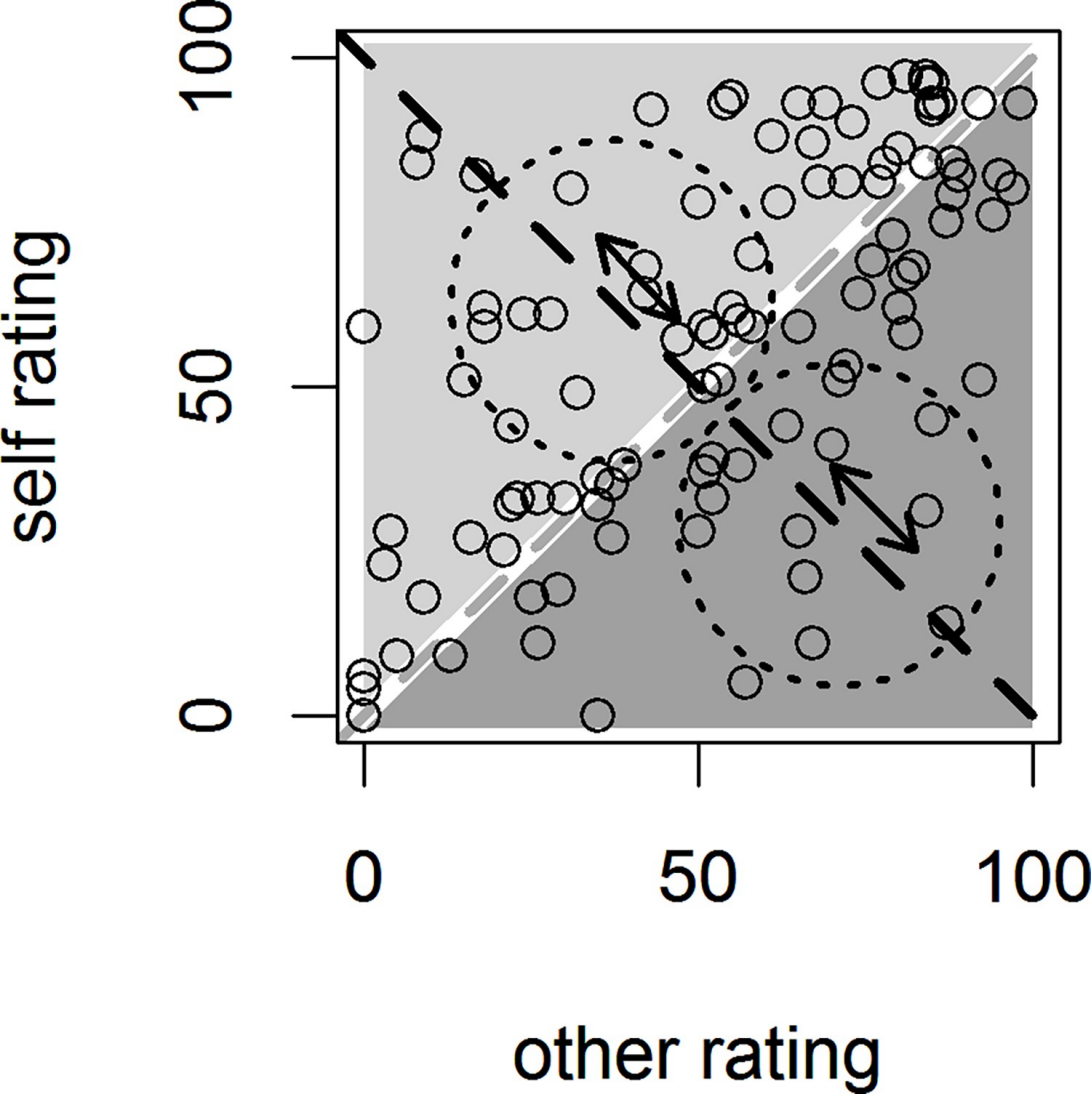
Rating space formed by a participant’s self- and other ratings. On the minor diagonal (light grey dashed line), self rating = other rating. Words in the light shaded area favor the participant, words in the dark shaded area favor the other. All rating differences, and thus the evidence e, are traversed by the major diagonal (black dashed line). Along this diagonal the two staircases move (doted circles).

The participant’s self- and other ratings guided which adjectives were presented on a trial. These standard ratings form the axes of a two-dimensional rating space containing all adjectives (see Fig 2). The evidence provided by a word can be quantified as:

e = self rating—other rating

All values of e are traversed by the major diagonal. To control difficulty, a staircase algorithm determined which adjectives to present per trial [4]. Two staircases were moving along the major diagonal, separately for the self- and other condition. Both staircases were capped at the lower and upper end of the evidence scale, i.e. -100 and 100. A condition’s staircase started at the position on the diagonal that corresponded to the median value of e of all words favoring the condition’s correct response. When a correct response was given, the respective staircase was moved one step in the direction favoring the incorrect response; when an error was made, the staircase was moved 2 or 3 steps in the direction favoring the correct response (alternating in the order 2-2-3). This ratio achieves an accuracy of around 0.7 in the two conditions. The step size for the two staircases was the range of e divided by 20.

To select adjectives, the staircase’s position was used as the center of a two dimensional Gaussian distribution in the rating space. The variance in both the self- and other dimension was initially set proportional to the distance between the starting values of the two staircases, covariances were set to zero. The circular probability function for selecting the adjectives was chosen so that the self- and other ratings of the words presented in a trial were not correlated. Each word’s probability of getting picked for the current trial was proportional to that word’s value under the Gaussian, adjusted for the density of words on the evidence scale. This was done to prevent overpopulated values of e from being overrepresented. Density was estimated using kernel density estimation provided by the MASS package in the R language [30]. A word could only appear once per trial. Because of the often non-uniform distribution of words across the rating space, using the same variance for the two dimensional Gaussians would often lead to different variances of the word’s ratings in the two conditions. To overcome this, the initial variance of the Gaussians was adjusted so that the variance of the words’ ratings in the self- and other condition were comparable. Finally, the four words drawn for a decision trial were shuffled in place so that each word-position pairing was equally likely.

### Software and data analysis

All statistical analyses were performed using R [31]. Psychometric functions (choice functions) were fit to the individuals’ data using R’s generalized linear model function *glm* with the logit link function specified. Beyond the built-in functionality, we used the *ez* package [32] to perform repeated measures ANOVAs and the *BayesFactor* package [33] to compute Bayes factors for ANOVA designs. Effect sizes for ANOVAs will be reported as generalized eta squared (*η*_*g*_*^2^*). The behavioral task was implemented via custom C code using the Simple Directmedia Layer Library [34], which in turn was called from the R environment. The task was displayed at 60 Hz with a screen resolution of 1280 x 720.

## Results

### Accuracy, uncertainty and confidence

**Fig 3:**
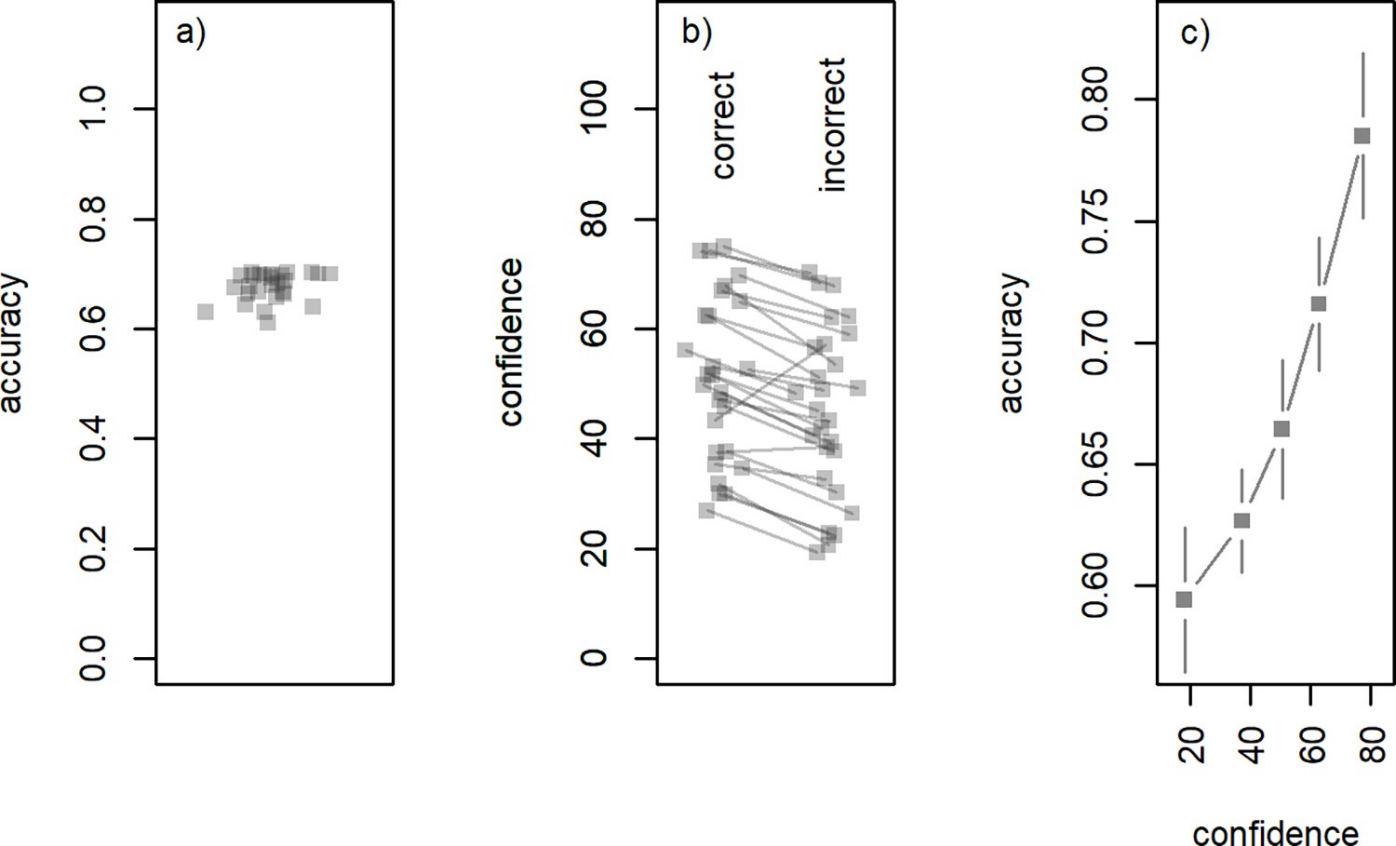
Accuracy and confidence during uncertain trait judgments. (A) Per-participant accuracy. (B) Per-participant average confidence for correct and incorrect responses. (C) Relationship between binned confidence and average accuracy, over all participants (vertical lines indicate 95% confidence intervals).

We first investigate whether our task was indeed able to create uncertain, yet precisely controlled trait judgment trials. When comparing participants’ responses about the trait adjectives to whether the trial belonged to the self- or other condition, choice accuracy was 0.68 (SD = .03, range = 0.61–0.7; see Fig 3A). Thus, trait judgments were indeed uncertain, very close to the intended 0.7 accuracy, and with relatively little variation among participants. When testing the participants’ accuracies against 0.7 in a one-sample t-test, the difference of around 2% was significant, t(26) = 4.73, p < .001.

While the task was able to control choice accuracy, it is also important that this control resulted in subjectively uncertain judgments for the participants. The average confidence rating over participants, on a scale of 0 to 100, was 49 (SD = 15). Participants’ confidence ratings distinguished between correct (M = 51, SD = 15) and incorrect (M = 45, SD = 15) choices (paired-sample t-test: t(26) = 6.36, p < .001). Only two participants did not show higher confidence for correct choices (see Fig 3B and 3C) shows the relationship between binned confidence judgments and accuracy, both averaged over participants.

### Choice functions

It has been repeatedly reported in perceptual choice tasks that for confident trials, the function relating stimulus strength to response probability is steeper than in non-confident trials, corresponding to a more efficient use of stimulus information in confident trials.

For each participant, every trial’s overall stimulus strength was computed as the average rating in favor of the trial’s target, over all four words, minus the average rating in favor of the non-target. The resulting ratings were converted to z-scores per participant. The more the word stimuli pointed toward the correct response, the more positive the stimulus strength. Per participant, we modelled the log-odds of correct responses as depending on the aforementioned stimulus strength as well as an intercept (i.e. the common logit regression model). This regression was performed per participant, separately for self- and other target trials and separately for low- and high confidence trials, determined via a per-participant median-split of the confidence judgment.

**Fig 4:**
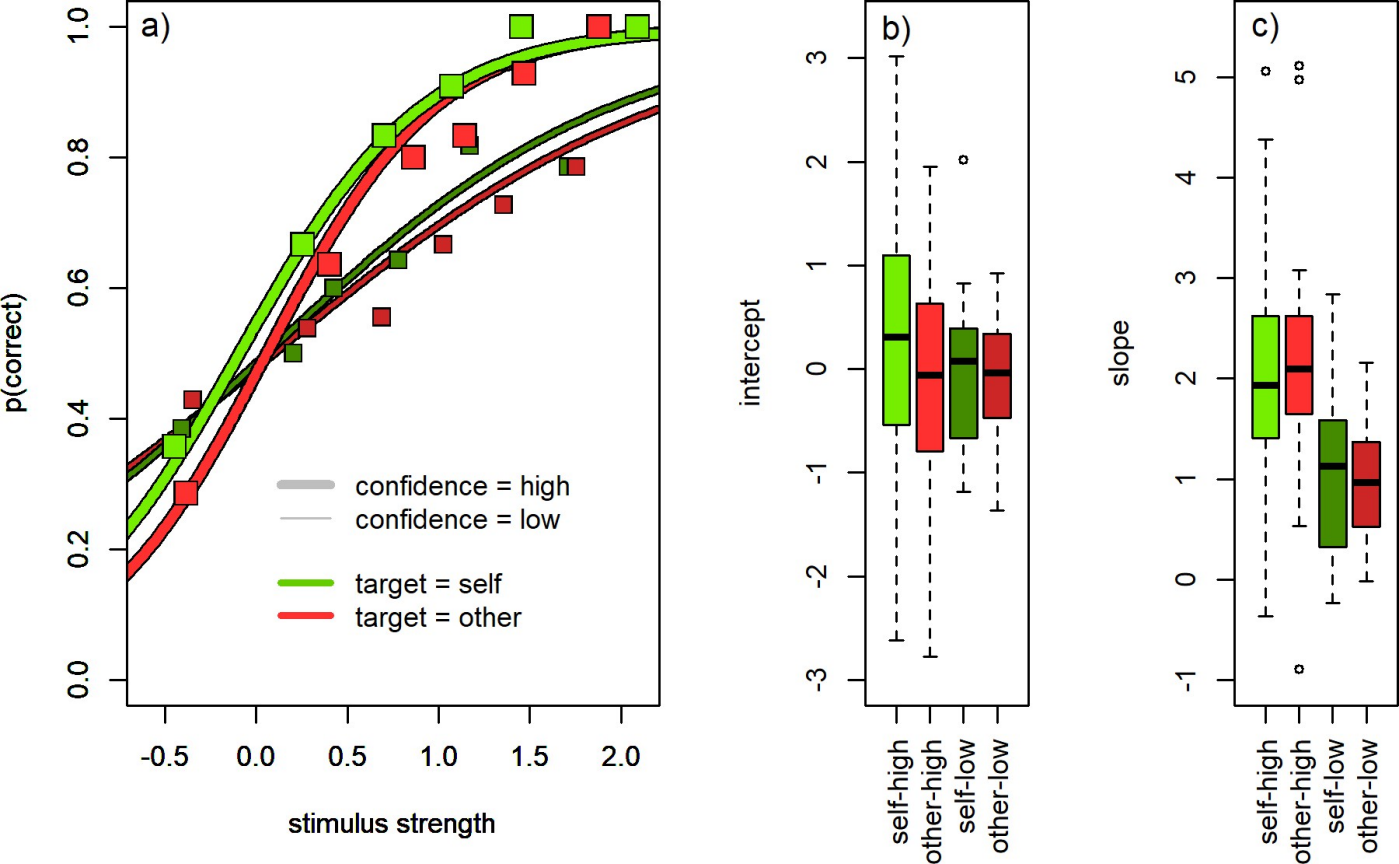
Psychometric function fits relating stimulus strength (target ratings–non-target ratings, standardized) to the probability of responding correctly, separately for self target trials (green) and other target trials (red) as well as high (light) and low (dark) confidence responses. (A) Mean psychometric functions constructed by averaging the intercepts and slopes from individual participants’ function fits (lines). Squares represent the median values of the binned behavioral data. (B) Boxplots illustrating the distribution of intercepts from the individual participants’ function fits. The box extends from first to third quartile, the central bar represents the median, whiskers cover the range, points represent outliers. (C) Same as b) but for the individual slopes.

Fig 4A shows the average psychometric functions of self- and other target trials, separately for trials with high- and low confidence responses. The functions were constructed by averaging the intercepts and slopes from the individual participants’ psychometric function fits. The mean intercepts of the four conditions, averaged over individual function fits, were–self target, high confidence: M = .18, SD = 1.17; other target, high confidence: M = -.12, SD = 1.24; self target, low confidence: M = -.06, SD = .73; other target, low confidence: M = -.09, SD = .64. The distributions of individual participants’ intercepts and slopes are illustrated using boxplots in Fig 4B and 4C. Individual psychometric functions are shown in the supplement (S1 Fig). None of the intercepts significantly deviated from zero in single-sample t-tests (all ps > .4), indicating that the probability of responding correctly was not significantly different from 0.5 for neutral stimuli with a signal strength of zero. The intercepts also did not differ between low- and high confidence trials in paired-sample t-tests–for other target, t(26) = 0.15, p = .88; for self target, t(26) = 1.38, p = .18. Neither did they differ between self- and other target trials–for low confidence trials, t(26) = .12, p = .91; for high confidence trials, t(26) = .69, p = .50. Thus, the base probability of responding correctly to neutral, non-informative stimuli did not differ between high- and low confidence trials, and not between self and other target trials.

The averaged slopes for stimulus strength were–self target, high confidence: M = 1.97, SD = 1.22; other target, high confidence: M = 2.13, SD = 1.20; self target, low confidence: M = 1.04, SD = .82; other target, low confidence: M = .92, SD = .53. Slopes were significantly steeper for high- compared to low confidence trials–self target trials: t(26) = 4.47, p < .001; other target trials: t(26) = 6.08, p < .001. Slopes were not different between self- and other target trials–for low confidence trials: t(26) = .79, p = .44; for high confidence trials: t(26) = .77, p = .45. The psychometric function relating stimulus to probability of responding correctly was thus steeper for high confidence trials.

### Reverse correlation

Per trial, the self ratings and other ratings of the four presented words influence the participant’s choice. This influence can be quantified via the method of reverse correlation [7]. Using this method, studies on perceptual decision making have found that binary choices are symmetrical, i.e. both evidence in favor of the chosen and non-chosen alternative affects choices, the former in a positive direction and the latter in a negative direction [8,10].

During reverse correlation, random stimulus fluctuations around the expected stimulus value are correlated to the participant’s responses. In our case, the random stimulus fluctuations were computed by taking the self- and other ratings of a trial’s four adjectives, and subtracting from them the expected ratings for that trial based on the staircase algorithm. These “raw” fluctuations were divided by their standard deviation over all trials. The result is referred to as the *decision weight* and quantifies the association between the words’ ratings and the participant’s response. Symmetrical choices are reflected by positive decision weights for the stimulus dimension that is chosen by the participant, and negative decision weights for the dimension not chosen. To compute the weights for the chosen- and non-chosen alternative, per trial the decision weights stemming from the self ratings and other ratings were re-labelled as chosen- and non-chosen based on the trial’s response. To perform reverse correlation of confidence judgments, the previous weights were computed again, but separately for high- and low confidence trials. We then subtracted the low confidence decision weights from the high confidence decision weights, resulting in difference weights that reflect how strongly the underlying signal affects participants’ confidence judgments [10]. We refer to these as **confidence weights.**

The symmetry of decision weights around zero was tested via two simultaneous criteria: first, whether the weights for the chosen and non-chosen stimulus dimension indeed differed from each other; and second, whether an inversion of the non-chosen weights removed this difference, which would suggest that non-chosen weights are indeed the mirror image of chosen weights. The second criterion comes down to a confirmation of a non-effect, which we evaluated in two ways: first, we judged the associated effect size in the repeated measures ANOVA to get an idea whether there might be an effect escaping the significance test due to insufficient power; second, we computed the associated Bayes factor to estimate whether the data present more evidence for the absence of an effect than for its presence.

**Fig 5:**
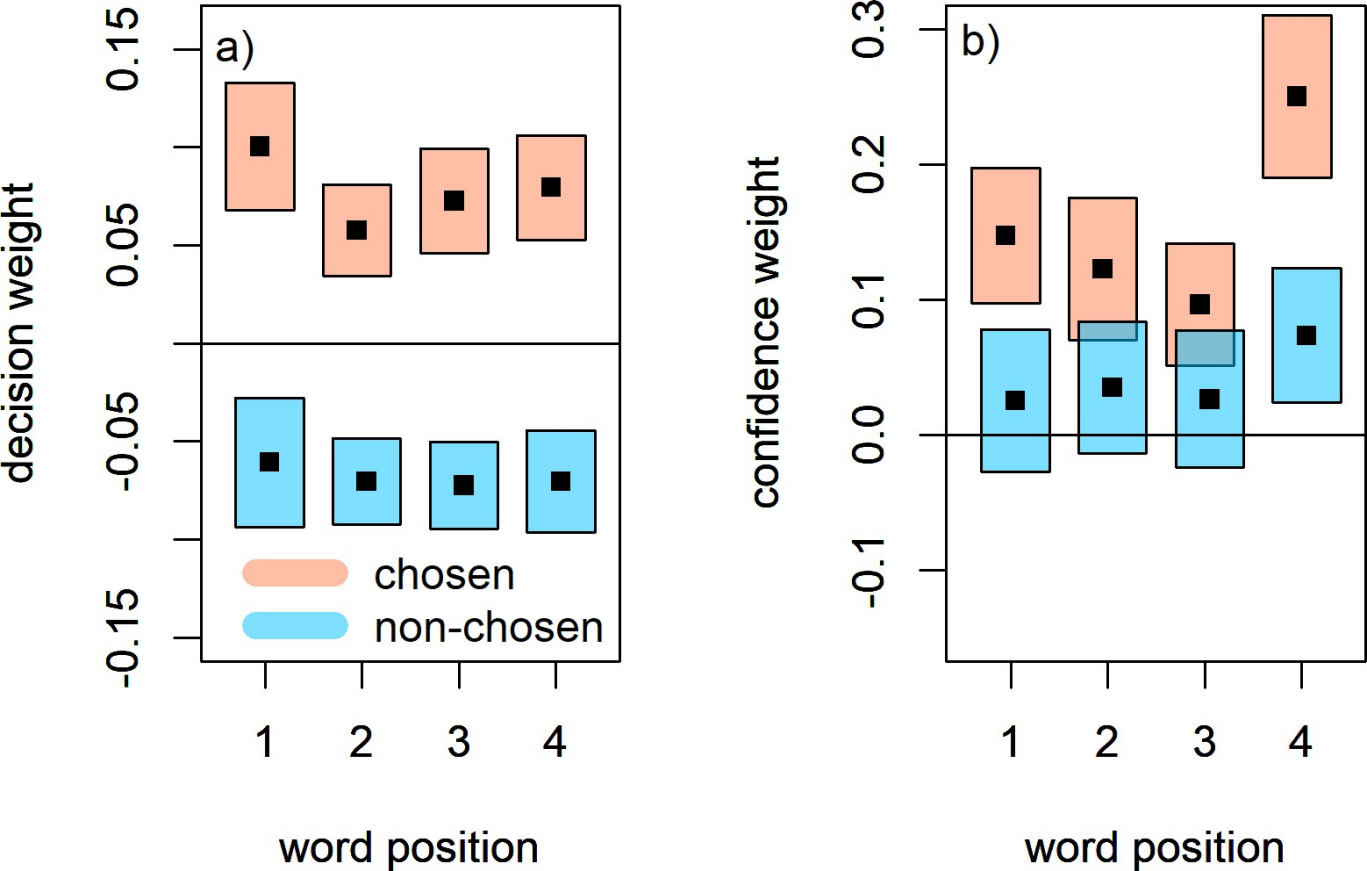
Reverse correlation results. (A) Decision weights, quantifying how fluctuations in the chosen stimulus dimension (red) and non-chosen dimension (blue) affect trait judgments. (B) Confidence weights, quantifying how stimulus fluctuations affect metacognitive judgments. Shaded areas indicate 95% confidence intervals.

For the first criterion, we subjected the decision weights to a repeated measures ANOVA with choice congruence (chosen, non-chosen) and word position (1 through 4) as within-subject factors. The critical effect of choice congruence was significant, F(1,26) = 71.69, p < .001, η_g_^2^ = .52. For the second criterion, we inverted choice-incongruent weights and repeated the ANOVA, which removed the critical effect of choice congruence, F(1,26) = 1.11, p = .3, η_g_^2^ = .004. The size of the original effect was very large, while the non-significant effect was of a size considerably below what is commonly considered small [35]. This points to symmetrical decision weights for the primary trait judgment (see Fig 5A). No other effects were significant in either analysis. To further test the second criterion, we computed the Bayes factors for the same ANOVA design, using the data with inverted choice-incongruent decision weights. The Bayes factor of the choice congruence effect consists of the ratio of evidence for a model including this effect to the evidence for a model with just the person-specific intercepts. This Bayes factor was BF_10_ = .26, pointing toward the model without congruence effect to be close to four times more likely, given the data.

The positive evidence bias during confidence judgments manifests itself in a clear asymmetry in confidence weights, with reduced or even zero weights for choice incongruent information, but positive weights for choice congruent information. We judged this pattern by two criteria: first, we tested whether confidence weights for choice incongruent stimulus information were smaller than for congruent information; second, at each word position, we tested whether confidence weights for incongruent stimulus information significantly deviated from zero.

The first test was performed by subjecting confidence weights to a repeated measures ANOVA, with choice congruence (chosen, non-chosen) and word position (1 through 4) as within-subject factors. The critical effect of congruence was significant, F(1,26) = 25.1, p < .001, η_g_^2^ = .15. The second test is performed via one-sample t-tests of non-chosen weights at each word position: for position 1, t(26) = .95, p = .35; for position 2, t(26) = 1.4, p = .17; for position 3, t(26) = 1.02, p = .32; for position 4, t(26) = 2.9, p = .007. Thus, there was a large bias during confidence judgments, such that the non-chosen stimulus information was severely discounted, with some residual influence especially of the final word in the four word sequence.

Besides the critical effect of congruence, there was also a significant main effect of word position (F(3,78) = 6.37, p < .001) as well as a significant interaction between congruence and word position (F(3,78) = 3.12, p = .031). The effect of word position mainly consisted of the aforementioned pattern of the final word exerting a stronger influence on confidence than the first three (see Fig 5B), which is reminiscent of recency effects during studies of recall. Indeed it seems plausible that after the tightly timed presentation of the word sequence, the final word was more present in memory and thus had a stronger effect on metacognitive judgments, especially since these second-order judgments happened after primary choices and were therefore more removed in time from stimulus presentation, forcing participants to rely on memory traces of the verbal stimuli.

## Discussion

We presented the results of a trait judgment task in which groups of adjectives had to be categorized as self- or other descriptive. We were able to control task difficulty by first acquiring per-word ratings of how well the item fits the two response options. These ratings also allowed us to apply quantitative methods of analysis, such as fitting psychometric functions and reverse correlation of responses to stimulus fluctuations.

When examining the overall difficulty of our trait judgment task, mean accuracy was 0.68, very close to the 0.7 intended by the staircase algorithm, with a standard deviation of 0.03. Broadly speaking, the method of controlling trait adjective presentation to create uncertainty in the judgment was successful. The slight deviation of 2% might be the result of the limited stimulus space visible in Fig 2: from this space, word stimuli were selected on a trial-by-trial basis. Toward the ends of the major diagonal, words become more strongly associated with one response, but they also become less abundant. Toward the middle of the major diagonal, words are more ambivalent with respect to who they describe best, but also more abundant. The staircase algorithm responsible for setting the difficulty is moving along this diagonal and, due to the differential availability of words, might be slightly biased toward more precise control when approaching the ambivalent part of the stimulus space, compared to the certain area. The resulting deviation is small but could likely be overcome by making stimulus selection narrower toward the outer edges of the stimulus space.

While our method of stimulus control can create an objectively uncertain judgment situation, as measured by choice accuracy, it is not guaranteed that participants also perceive this as a partially uncertain decision. After all, we define accuracy with respect to a separate standard rating that is also subjective and prone to measurement error, and it is possible that participants in fact either felt that choices were completely certain, or conversely, felt that choices were wholly random. In this case, our conception of accuracy would not match the psychological experience of uncertainty. However, the confidence judgments gathered after each decision show that indeed participants felt partially unsure about their choices. Furthermore, subjective confidence was clearly associated with accuracy, as evidenced by the fact that correct trials included higher confidence judgments (Fig 3B) and that confidence and accuracy co-varied in a graded fashion (Fig 3C). Thus, our method of stimulus presentation was able to control accuracy as well as create subjectively uncertain choice situations in which meaningful confidence judgments could be elicited.

Since experimental stimulus control appears to have been largely successful, we used the standard word ratings to quantitatively analyze the participants’ responses. We first fit logistic models predicting the probability of responding correctly from the amount of information given by the word stimuli. This analysis is analogous to typical evaluations of response accuracy as a function of stimulus strength in perceptual decision tasks. Using this method, we replicated a common effect from more basic choice tasks, namely that high confidence trials showed steeper psychometric functions than low confidence trials. The slope of these functions is often interpreted as the efficiency with which the cognitive system processes stimulus information, and our finding fits the idea that more efficient stimulus use results in higher confidence. The fact that this previously reported effect could be replicated in the current trait judgment task suggests that the method of controlling verbal stimulus presentation via separately assessed standard ratings is able to capture basic properties of the process of personality perception and decision.

The fact that the psychometric function can describe the relationship between stimulus information and response probability well in the current task has important methodological implications. For example, it allows using more refined stimulus presentation schemes from the psychophysical literature. Methods like the PSI algorithm for stimulus presentation [5] explicitly estimate the parameters of the (parameterized) psychometric function underlying a participants performance, while the task is ongoing, in order to place to next stimulus at the maximally informative level. This and similar methods [6,36] can be used to increase the efficiency of the present type of trait judgment task. Furthermore, the abstraction of measured performance via the psychometric function allows characterizing participants in a limited number of parameter values (midpoint, slope, etc.), which can be used for further analysis.

An interesting incidental finding is the fact that the psychometric functions fit to participants’ response data did not show evidence of a bias in favor of responding “self” or processing self related information more efficiently during uncertain trait judgments. These properties of choices would have been captured in the functions’ intercepts and slopes being increased in the self condition’s trials, relative to the other condition. This finding might be somewhat surprising given various models and reports of self-prioritization in the social cognition literature [37–41]. According to many of these models, when a stimulus makes reference to a person’s self concept, this affords it a processing advantage. The most well-known example of this is the cocktail party effect [38]. Similarly, it could have been hypothesized that when deciding whether an ambivalent group of trait adjectives describes oneself or another person, participants might be more sensitive to the degree to which the words match their self concept, compared to how well it matches the other person, or might show a general tendency toward judging words as self descriptive more often than judging them as other descriptive. This in turn would have resulted in either a steeper slope for the choice functions in the self condition, or a higher intercept.

We can only speculate as to the reasons why no such effects of self prioritization were found in our task. A possible explanation for the lack of an intercept effect between the self and other condition might be the phenomenon of probability matching [42]. Probability matching describes the tendency of participants’ response proportions to approximate the probability of these responses being correct. If this explanation is correct, participants might naturally show an elevated probability of judging words as self descriptive, but make their responses in our two alternative choice task conform to the assumed equal proportion of self- and other target trials. If probability matching indeed contributes to this lack of an effect on intercepts, manipulations known to affect matching (e.g. feedback) might make effects of self prioritization visible.

An important methodological issue for the current method of presenting verbal choice stimuli is that when interpreting behavioral results, it should always be kept in mind that the original, participant provided standard ratings are likely not absolutely precise, and might indeed be biased. The issue of limited precision of standard ratings is important but relatively straightforward, as it simply puts an upper limit on the precision of all analyses and manipulations that are based on these ratings. This problem is also open to further methodological refinement, since the ratings’ precision can be estimated and improved by gathering multiple ratings per adjective. The issue of biased standard ratings is more complex, because without an additional, more objective external criterion of self- and other person fit, this bias cannot be assessed. For example, during the initial adjective rating task a participant might give systematically inflated ratings of how well the words fit his/her own personality (i.e. words that fit the other person somewhat better might nevertheless be rated as fitting both the other and the participant to the same degree). Because subsequent analysis of responses is based on these biased ratings, the researcher will be blind to this same bias during responding (i.e. the participant might judge groups of apparently neutral adjectives as self-descriptive in 50% of trials–thus appearing unbiased–when in fact these adjectives are more descriptive of the other person, and the participant’s responses are “self biased”).

Potentially biased ratings have to be considered when interpreting patterns of trait judgments, but in our view these cautionary notes do not invalidate the method and task. First, this same problem also exists for studies on value-based decision making using similar standard rating methods [19,20], and indeed it could be said that every time the relationship between two subjective variables gathered from a participant is investigated, one or both could well be biased. Second, not all choice effects are readily explained by biased standard ratings. The fact that we replicated several common effects from studies on perceptual decision making suggests that meaningful results can indeed be obtained. Third, stimulus ratings need not be limited to the participants themselves. For example, controlled trait judgment tasks such as the one presented here might also be gathered in dyads of participants, with each person only providing adjective ratings for the other person, but stimulus presentation during the tasks controlled by both the participants’ ratings. In this case, biased self ratings could be excluded as an alternative explanation. More generally, methods that are known to reduce judgment bias can be applied when gathering within-study stimulus ratings, such as financial incentives or more objective types of assessment instruments.

When analyzing the influence of self- and other ratings using reverse correlation, we found that primary trait judgments are symmetric while confidence judgments are almost exclusively affected by information for the selected option. This metacognitive bias has been reported previously in a number of studies (also known as positive evidence bias; [e.g. 10,14,25]), and in the present work, serves as evidence that reverse correlation analysis can be successfully applied to a task of trait judgments in order to elucidate which stimulus aspects affect choice and confidence during personality judgments.

The fact that stimulus influence on personality judgments can be investigated in this way opens up interesting avenues for research on the cognitive processes of personality perception. For example, psychology has a long history of searching for the basic factors underlying our conception of personality [43], and taxonomies such as the Big-Five dimensions are often discussed. If these factors indeed form the basic language in which we construct and perceive personality, it will be interesting to see if this structure can also be discovered in reverse correlation profiles of uncertain personality trait judgments. In this case we would expect traits that are clearly aligned with major dimensions of personality (e.g. *outgoing* related to *extraversion*) to have a stronger influence on trait judgments than less aligned traits (e.g. *moral*). Other interesting topics of personality perception that lend themselves to investigation via tasks of uncertain trait judgments are the effects of reflected appraisals compared to non-reflected appraisals [22] or the influence of the degree of closeness between two people on how self-other trait judgments are performed.

## Supporting information

S1 Fig Individual choice function fits. The relationship between stimulus strength (x) and probability of responding correctly (y), as captured by the psychometric functions we fit to each individual participant (see section Methods–Choice functions). Function fits are illustrated separately for the self target condition (b, d) and other target condition (a, c), as well as low- (a, b) and high confidence trials (c, d). Thick lines indicate average psychometric functions (as in Fig 4A).
(TIF)
