# Supplementary figures and images for: Symmetrical choices and biased confidence during uncertain personality trait judgments
Source: PLoS One. 2024 Oct 31;19(10):e0312858. doi: 10.1371/journal.pone.0312858 (PMC11527456; doi:10.1371/journal.pone.0312858)

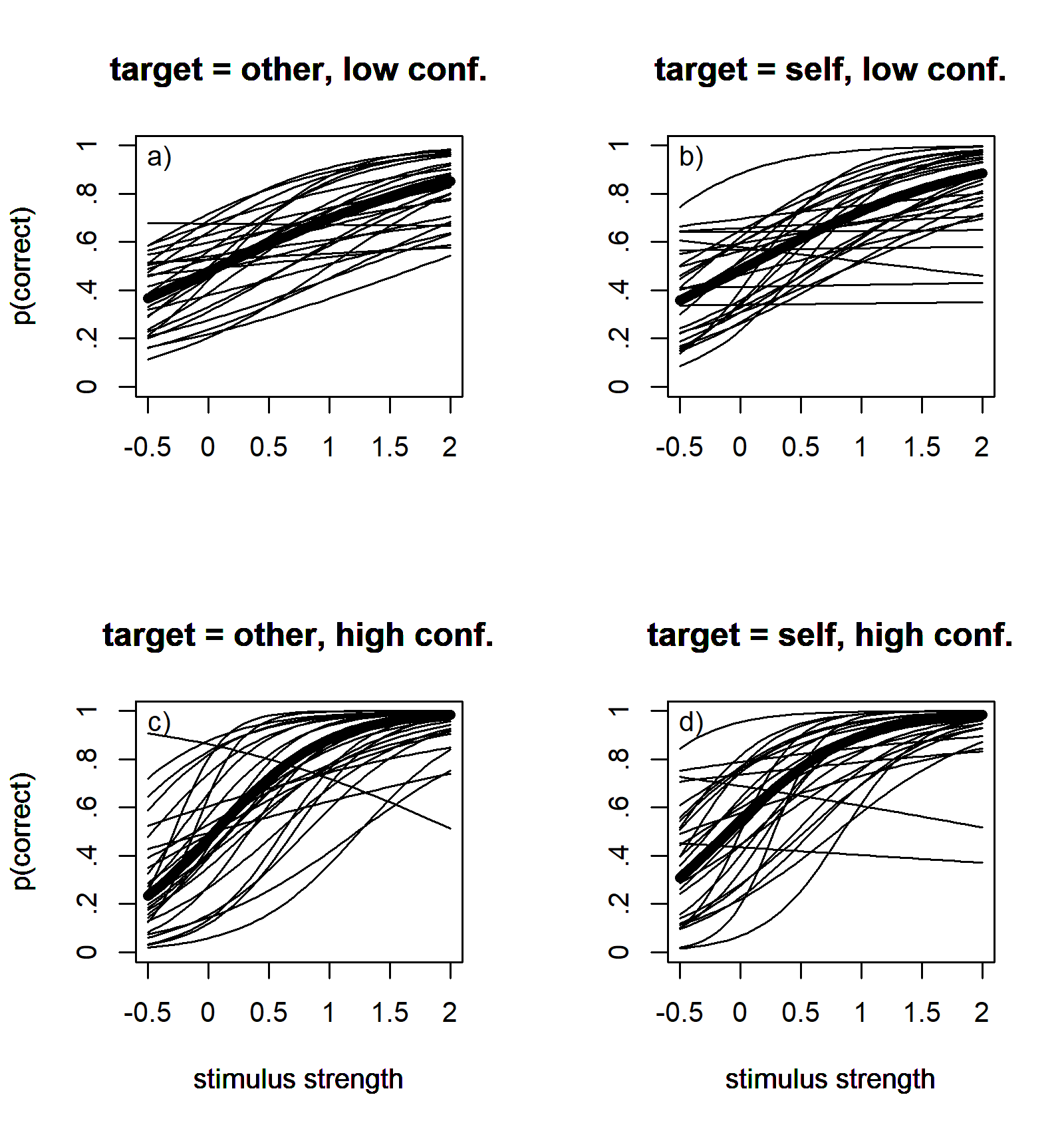

Supplement: S1 Fig — The relationship between stimulus strength (x) and probability of responding correctly (y), as captured by the psychometric functions we fit to each individual participant (see section Methods–Choice functions). Function fits are illustrated separately for the self target condition (b, d) and other target condition (a, c), as well as low- (a, b) and high confidence trials (c, d). Thick lines indicate average psychometric functions (as in Fig 4A). (TIF) [file pone.0312858.s001.tif]
